# Fine Particulate Matter and Respiratory Healthcare Encounters among Survivors of Childhood Cancers

**DOI:** 10.3390/ijerph16061081

**Published:** 2019-03-26

**Authors:** Judy Y. Ou, Heidi A. Hanson, Joemy M. Ramsay, Claire L. Leiser, Yue Zhang, James A. VanDerslice, C. Arden Pope, Anne C. Kirchhoff

**Affiliations:** 1 Huntsman Cancer Institute, University of Utah, Salt Lake City, UT 84112, USA; Heidi.Hanson@hci.utah.edu (H.A.H.); Joemy.Ramsay@hci.utah.edu (J.M.R.); Anne.Kirchhoff@hci.utah.edu (A.C.K.); 2 Department of Internal Medicine, Division of Epidemiology, University of Utah, Salt Lake City, UT 84132, USA; Zhang.Yue@hsc.utah.edu; 3 Department of Surgery, University of Utah, Salt Lake City, UT 84132, USA; 4 Population Sciences, Huntsman Cancer Institute, University of Utah, Salt Lake City, UT 84112, USA; Claire.Leiser@hci.utah.edu; 5 Department of Family and Preventive Medicine, Division of Public Health, University of Utah, Salt Lake City, UT 84108, USA; Jim.Vanderslice@utah.edu; 6 Department of Economics, Brigham Young University, Provo, UT 84602, USA; Cap3@byu.edu; 7 Department of Pediatrics, University of Utah, Salt Lake City, UT 84108, USA

**Keywords:** air pollution, children, cancer survivorship, late effects

## Abstract

Some chemotherapies that treat childhood cancers have pulmonary-toxic properties that increase risk for adverse respiratory-health outcomes. PM_2.5_ causes similar outcomes but its effect among pulmonary compromised cancer survivors is unknown. This case-crossover study identified the PM_2.5_-associated odds for primary-respiratory hospitalizations and emergency department visits among childhood cancer survivors in Utah. We compared risk among chemotherapy-treated survivors to a cancer-free sample. We calculated 3-day-average PM_2.5_ by ZIP code and county for event and control days. Conditional logistic regression estimated odds ratios. Models were stratified by cause of admission (infection, respiratory disease, asthma), previous chemotherapy, National Ambient Air Quality Standard (NAAQS), and other variables. Results are presented per 10 µg/m^3^ of PM_2.5_. 90% of events occurred at 3-day PM_2.5_ averages <35.4 µg/m^3^, the NAAQS 24-h standard. For survivors, PM_2.5_ was associated with respiratory hospitalizations (OR = 1.84, 95% CI = 1.13–3.00) and hospitalizations from respiratory infection (OR = 2.09, 95% CI = 1.06–4.14). Among chemotherapy-treated survivors, the PM_2.5_-associated odds of respiratory hospitalization (OR = 2.03, 95% CI = 1.14–3.61) were significantly higher than the cancer-free sample (OR = 0.84, 95% CI = 0.57–1.25). This is the first study to report significant associations between PM_2.5_ and respiratory healthcare encounters in childhood cancer survivors. Chemotherapy-treated survivors displayed the highest odds of hospitalization due to PM_2.5_ exposure and their risk is significantly higher than a cancer-free sample.

## 1. Introduction

Fine particulate (PM_2.5_) pollution is linked to respiratory infections and is capable of exacerbating preexisting respiratory conditions among vulnerable children and adults [1,2,3,4,5,6,7]. Similar to children with preexisting conditions, survivors of childhood cancers may experience increased vulnerability to PM_2.5_ due to lung damage and potential immunosuppression resulting from cancer and treatment with chemotherapy [8,9]. Respiratory conditions resulting from the chemotherapy used to treat cancer can start within the initial months after treatment ceases [10,11,12,13,14,15,16]. These conditions include respiratory infection, difficulty breathing, chronic coughing, and asthma. Respiratory conditions are a primary cause of non-cancer mortality in survivors of childhood cancers who are a few years to a few decades off therapy [17]. Respiratory infections, such as pneumonia and bronchitis, are a particular concern in this population as survivors of childhood cancers face a significantly higher risk for infection than siblings without a cancer history [9,14,18]. 

Respiratory infections and conditions are also linked to PM_2.5_ pollution exposure nationwide and in the state of Utah [1,2,3,4,19,20]. Utah’s most populated counties have severe chronic and short-term air pollution problems that have been linked to respiratory problems in the general Utah population [19,21,22]. PM_2.5_ in Utah has specifically been linked to acute lower respiratory infection among children, adolescents, and young adults, and to pneumonia in adults aged ≥65 years [19,20]. Utah’s PM_2.5_ originates largely from traffic emissions [23], but winter inversions further expose Utah’s population to 24-h PM_2.5_ concentrations as high as 70 µg/m^3^ [24], roughly six times the 24-h national health standard [25]. As a result Utah has some of the worst short-term PM_2.5_ pollution in the nation [26]. 

To the best of our knowledge, no studies have examined the short-term effects of PM_2.5_ pollution on the respiratory health outcomes of childhood cancer survivors despite their propensity for respiratory problems. To address this gap, we conducted a case-crossover study to examine the effect of short-term PM_2.5_ exposure on respiratory hospitalization and emergency department (ED) visits, separately, among survivors of childhood cancers. We identified hospitalizations and ED visits using statewide healthcare encounter data from the Utah Population Database (UPDB). We also identified survivors’ PM_2.5_-associated risk for any respiratory infection by aggregating hospitalizations and ED visits with respiratory infection as the primary cause of admission. To identify whether receipt of chemotherapy during cancer treatment further modifies risk, we compared the PM_2.5_-associated risk for respiratory events among chemotherapy-treated survivors to the risk in a cancer-free sample.

Current regulatory guidelines for air pollution may not be sufficient to protect vulnerable groups such as cancer survivors [27]. To understand whether cancer survivors are affected by PM_2.5_ pollution at sub-regulatory levels, we examined the risk for respiratory hospitalizations and ED visits by the 24-h PM_2.5_ National Ambient Air Quality Standard (NAAQS) of 35.4 µg/m^3^ and by 25.0 µg/m^3^, which was identified as a threshold for respiratory effects among an elderly population [27].

## 2. Materials and Methods

### 2.1. Procedures

Case-crossover studies are well-suited to study the effects of short-term PM_2.5_ exposure on acute respiratory events [28,29]. In the case-crossover design, subjects who experienced the outcome (event days) are identified, and information about each subject’s exposure during the time prior to the event is compared with that same individual’s exposure at other times (control days). Thus, the units of analysis for case-crossover studies are the event and control days, not individuals. The self-matching also nets out confounding from variables that do not change over time (e.g., sex, race). These variables can still be examined as effect modifiers [30]. The design requires that models control for variables that change daily (e.g., temperature). Sub-group specific effects for time-independent variables are determined through stratified models and comparisons of these stratum-specific estimates to determine effect modification can be achieved using an interaction term.

This case-crossover study was nested in a cohort used in previous studies of Utah childhood cancer survivors [10,31,32]. From this cohort we identified 3819 survivors who were diagnosed with a childhood cancer at age ≤25 years from 1 January 1986 and 31 December 2012, diagnosed or treated at Primary Children’s Hospital (PCH) in Salt Lake City, Utah, and alive ≥5 years from diagnosis. Since Primary Children’s Hospital is the only pediatric oncology clinic in the state of Utah, the hospital serves between 82% and 97% of childhood cancer patients in the state, depending on age at diagnosis, although patients aged ≥15 years are also often treated by adult hospitals [33,34]. Thus, our cohort represents the vast majority of childhood cancer patients and survivors diagnosed under the age of 15 in the state [10,31,33]. We excluded cancer survivors diagnosed with epithelial neoplasm according to the International Classification for Childhood Cancer definition. The University of Utah Institutional Review Board approved this study. 

### 2.2. Event Day Selection

We defined event dates as any respiratory hospitalization occurring between 1 January 1996 and 31 December 2013 or respiratory ED visit between 1 January 1996 and 31 December 2015 that occurred ≥5 years post-diagnosis. If a relapse occurred, we included events between the date ≥5 years after first diagnosis and the date of relapse, as ascertained from patient medical records.

We obtained statewide hospitalization discharge data from the UPDB, which links to Utah Department of Health records. Dates of ED visits were found using the Intermountain Health (IH) and the University of Utah data warehouses. These two systems serve >80% of the state [35]. 

### 2.3. Control Day Selection

We used a bidirectional time-stratified [28] approach to select control days that were in the same month as the event day and occurred 7, 14, and 21 days before and after the event day [28]. This resulted in 3 to 4 control days per event day.

### 2.4. Event and Control Days in a Cancer-Free Sample

To compare estimates to a cancer-free sample, we identified hospitalizations and ED visits nested within a previously established cancer-free sample of the Utah population [10,31,32]. Event and controls days for this sample had the same entry criteria as the event and controls days for survivors. The ratio of events in the cancer-free sample to events in the survivor sample was 1.2:1 for hospitalizations and 2:1 for ED visits. 

### 2.5. PM_2.5_ Exposure Assessment

PM_2.5_ was measured at the ZIP code and county-level depending on years of data availability. ZIP code-level measurements were used to measure PM_2.5_ exposure from 1999 to 2015 while county-level PM_2.5_ estimated using daily PM_10_ values were used to measure exposure during the years 1996 to 1998. We obtained residential ZIP codes from each hospital and ED record. 

From 1999 to 2015 we estimated daily PM_2.5_ levels for the 2010 population-weighted centroid of each residential ZIP code using data from the U.S. Environmental Protection Agency Datamart, which contains daily PM_2.5_ measurements for the entire nation, including the state of Utah [36]. Using topographic features, we delineated 20 air basins located across the state. Air basins were defined as areas where lateral air movement would be reduced due to mountain ranges. Six of the air basins were in four Utah counties that contain 80% of Utah’s population and major cities, including Salt Lake City and adjacent large urban areas [37]. As monitoring stations are located all over the state, we assigned each monitor to the air basin where it was located using ArcGIS © version 9.3 (Esri, Redlands, CA, USA). We estimated daily PM_2.5_ for each ZIP code centroid using inverse distance weighting of all observations from monitoring stations located in the same air basin as the ZIP code centroid. The benefit of this method is that we were able to assign values by ZIP code, rather than county-level measurements from the raw data. If daily PM_2.5_ values for ZIP codes were missing, we substituted the daily county-level average. 

From 1996 to 1998, PM_2.5_ measurements were not available by ZIP code or for the entire state of Utah. For these years, we estimated daily county-level PM_2.5_ using no intercept regression models correlating PM_10_ and PM_2.5_ while accounting for stagnation [38]. Imputed data accounted for 10% of daily PM_2.5_ measurements in the four counties containing 80% of Utah’s population (Salt Lake, Utah, Davis, Weber).

For the analysis, PM_2.5_ exposure was calculated as a 3-day average PM_2.5_ (lag day 0 to lag day 2) based on a biologically relevant time period [39] and values were scaled by a unit of 10. We controlled for county-level temperature using data from the National Weather Service and Federal Aviation Administration [40]. 

### 2.6. Variables Used in Stratified Analyses

We used the following primary ICD-9 codes to categorize the cause of respiratory hospitalization or ED admission: Respiratory infection (460–465, 480–488, 466, 517.1, and 519.8); Respiratory disease (470–478, 490–494, 496, 514–516, 518.2–518.3, 510, 513, 511, 512, 517.2–517.8, 518.0–518.2, 518.8, 519.1–519.9); Asthma (493). We excluded events related to ambulatory surgeries or procedures, surgical complications, pregnancy, or infection from inhaled food/drink.

Other measures included cancer diagnosis and age at diagnosis obtained from IH records. Diagnosis date was used to calculate years since diagnosis (5 to 9 years vs. 10 to 29 years). We obtained race/ethnicity from the UPDB to examine as a potential effect modifier [41]. Chemotherapy, radiation, and surgery were ascertained from IH records and the Utah Cancer Registry (Yes/No).

### 2.7. Statistical Analyses

We limited the age of hospitalizations and ED visits to age <40 years in both the survivor and cancer-free sample to ensure comparability of events between the two groups. Thus, events occurred in the same age groups and in the same time frame in both groups. The vast majority of all hospitalizations and ED visits identified among survivors (94%) and the cancer-free sample (90%) occurred prior to age 40 years. We present hospitalizations and ED visits as separate outcomes. If any event occurred within two days of another event (e.g., two ED visits in two days), we removed the second visit (<2% of ED visits, <1% of hospitalizations). Since we reported hospitalizations and ED visits as individual outcomes, if an ED visit led to a hospitalization (*n* = 25) we counted the event as an ED visit and hospitalization separately. Subjects could contribute multiple hospitalizations or ED events. If a subject was hospitalized for multiple days, it was counted as a single event.

We ran conditional logistic regression models to calculate adjusted odds ratios, controlling for county-level temperature which varies widely due to Utah’s snowcapped mountains and hot desert environments [19,42]. Models were conditioned on subject to account for multiple events per subject, which increased power and reduced bias from inclusion of first event only [43]. If an event day was missing PM_2.5_, its associated event days were also excluded. This resulted in <0.5% of all event and control days excluded from analyses. 

We stratified models by cause of admission, previous chemotherapy, age at diagnosis, and years from diagnosis (5–9 years, 10–29 years) to obtain odds ratios for the effect of PM_2.5_ on the outcomes in these subgroups. These subgroups were chosen because the effects of PM_2.5_ might vary by these factors [10,11,12]. We also stratified odds ratios by cancer diagnosis.

Additional analyses examined associations by the 24-h NAAQS air quality standard of 35.4 µg/m^3^ and a lower level of 25.0 µg/m^3^ used in previous literature [27]. The 3-day average PM_2.5_ exposure for the days prior to hospitalization or ED visit were categorized as <35.4 µg/m^3^ or ≥35.4 µg/m^3^ and <25.0 µg/m^3^ or ≥25.0 µg/m^3^. Analyses were conducted by stratifying models by the two dichotomous categories. 

As respiratory infections are linked to PM_2.5_ exposure (19–21), we examined risk for all respiratory infections by combining hospitalizations and ED visits with primary ICD-9 codes for respiratory infection as a single outcome. Hospitalizations and ED visits that occurred on the same day were counted as a single event. These analyses were stratified by race/ethnicity, previous chemotherapy, and age at diagnosis. Statistical significance of the effect modification by race/ethnicity was determined using White survivors as the reference.

Models were run separately to identify the effect of PM_2.5_ on respiratory hospitalizations and respiratory infection for the cancer-free sample and survivor subgroups by treatment. We combined the groups in a single regression model with an interaction term to determine the statistical significance of the differences in effect with the cancer-free sample as the reference group [44]. 

Estimates were significant if p-value was <0.05. All results are reported per 10 µg/m^3^ unit increase in PM_2.5_. Results for hospitalizations and ED visits as a combined outcome are available in Appendix A. For this analysis, ED visits leading to hospitalizations were only counted once.

## 3. Results

Of the 3,815 eligible survivors, 185 subjects had a total of 335 respiratory events that met our eligibility criteria. We identified 68 respiratory hospitalizations and 267 ED visits among childhood cancer survivors (Table 1). Although our data included events that occurred across the entire state, 91% of hospitalizations and 75% of ED visits included in the final analysis took place along the Wasatch Front counties of Salt Lake, Davis, Utah, and Weber where the majority of the Utah population resides.

The mean 3-day average PM_2.5_ for all days was 10.0 of µg/m^3^ (range 1.15–68.7 µg/m^3^). The vast majority of hospitalizations (99.0%) and ED visits (96.4%) occurred at 3-day average PM_2.5_ levels <35.4 μg/m^3^ (not shown in tables), the national 24-h standard [45]. The mean age for hospitalizations was 21 years and the mode was 8 years; the mean age of ED visits was 22 years and the mode was 9 years. Respiratory infections were the primary cause of the majority of hospitalizations and ED visits. Most events came from White, Non-Hispanic survivors (82.1%) and survivors who received chemotherapy (59.7%) (Table 2). The events from Hispanic/other survivors largely came from Hispanic survivors (67.0%). Events most commonly arose from survivors of leukemia (26.5%) and lymphomas (28.1%) and survivors diagnosed age ≤3 years (29.4%). As the number of hospitalizations and ED visits in each cancer diagnosis group were small, we did not find significant or meaningful variation in the effect estimates by cancer diagnoses and did not provide these results for hospitalizations or ED visits.

### 3.1. Respiratory Hospitalizations

In Table 3, each 10 µg/m^3^-unit increase in PM_2.5_ was associated with an increased odds ratio (OR) for respiratory hospitalization (OR = 1.84, 95% CI = 1.13–3.00) among survivors. In stratified models, the PM_2.5_-associated odds for respiratory infection as a cause of hospitalization (OR = 2.09, 95% CI = 1.06–4.14) was significant. Due to low numbers, we did not report odds ratios for asthma hospitalizations. The PM_2.5_-associated odds of hospitalization among Hispanic survivors were elevated but not significant with a wider confidence interval, (OR = 2.22, 95% CI = 0.93–5.27), which may also be due to small numbers. The odds of hospitalization attributed to PM_2.5_ were significant among survivors who were given chemotherapy (OR = 2.03, 95% CI = 1.14–3.61) and survivors 5 to 9 years from diagnosis (OR = 1.75, 95% CI = 1.02–3.01). We found positive but non-significant odds for PM_2.5_ and respiratory hospitalizations among survivors diagnosed at age ≤3 years and survivors living 10 to 29 years from diagnosis. We examined the association of PM_2.5_ and respiratory hospitalization using the 24-h NAAQS standard of 35 µg/m^3^. For 3-day averages <35.4 µg/m^3^, we found the PM_2.5_-associated odds of hospitalizations was a marginally non-significant 1.79 (95% CI = 0.99–3.26). For 3-day averages <25 µg/m^3^, the PM_2.5_-associated odds of 2.48 was significant (95% CI = 1.16–5.05). The number of hospitalizations occurring at ≥35.4 µg/m^3^ and ≥25.0 µg/m^3^ were too small to yield stable effect estimates.

### 3.2. Respiratory Emergency Department Visits

Few odds ratios for the association of PM_2.5_ and respiratory ED visits were significant (Table 4). For survivors diagnosed at age ≤3 years, the PM_2.5_-associated odds of an ED visit was significant (OR = 1.58, 95% CI = 1.09–2.30). The association of PM_2.5_ and asthma-ED visits was positive but nonsignificant (OR = 1.17, 95% CI = 0.74–1.84). The non-significant PM_2.5_-associated odds of respiratory ED visit were slightly lower when the 3-day average PM_2.5_ was ≥35.4 µg/m^3^ (OR = 1.11) than when the average was <35.4 µg/m^3.^ (OR = 1.15). We found a similar pattern for the odds when examined by levels above and below 25.0 µg/m^3^. None of the odds ratios in these NAAQS categories were significant.

### 3.3. Respiratory Infections

For respiratory infections derived from combining hospitalizations and ED visits (Table 5), we found a significant association between PM_2.5_ and respiratory infection among cancer survivors of Hispanic/Other races (OR = 1.61, 95% CI = 1.04–2.49). The odds among Hispanic/Other survivors was marginally higher than the odds among White survivors (*p* = 0.06). The PM_2.5_-associated odds of respiratory infection was significant among survivors diagnosed at age ≤3 years (OR = 1.63, 95% CI = 1.03–2.58). We found a positive nonsignificant association for the PM_2.5_-rORelated odds among survivors treated with chemotherapy (OR = 1.24, 95% CI = 0.92–1.67).

### 3.4. Comparison of Odds among Chemotherapy-Treated Survivors, Surgery or Radiation Only Survivors, and a Cancer Free-Sample

**Figure 1:**
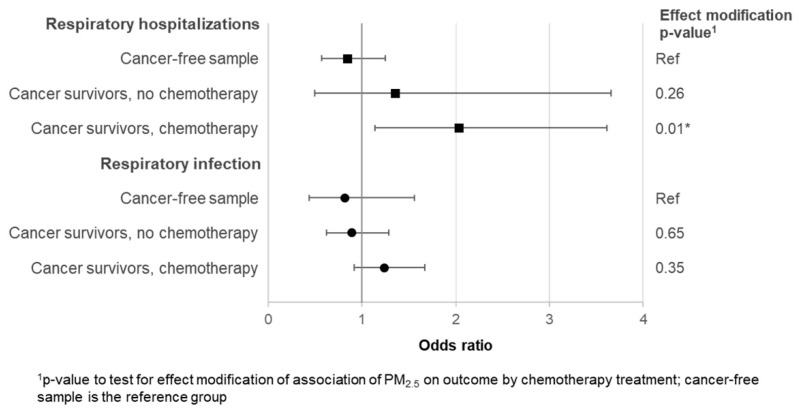
Comparison of odds ratios and 95% confidence intervals (CI) for the association of PM_2.5_ and respiratory events between survivors of childhood cancers and a cancer-free sample.

We found evidence of effect modification for the association of PM_2.5_ and respiratory hospitalizations by chemotherapy (Figure 1). Compared to the cancer-free sample (OR = 0.84, 95% CI = 0.57–1.25), the effect estimate for chemotherapy-treated survivors (OR = 2.03, 95% CI = 1.14–3.61) was significantly higher (*p* = 0.01). The difference between the cancer-free sample and the survivors treated with surgery and/or radiation alone (OR = 1.35, 95% CI = 0.50–3.66) was not significant. The odds for the effect of PM_2.5_ on respiratory infection (combined outcomes) were higher among the chemotherapy-treated survivors than either the surgery and/or radiation alone group (OR = 0.89, 95% CI = 0.62–1.29) or the cancer-free group (OR = 0.82, 95% CI = 0.44–1.56), but the p-value for the test of effect modification was not significant.

## 4. Discussion

The current population of 429,000 cancer survivors diagnosed in childhood in the United States (US) is projected to grow with advances in diagnostic and treatment technology [46]. These cancer survivors may also live in one of the 40% of communities in the US with poor air quality [47]. The long-term effects of cancer treatment cannot be reversed but studying how air pollution affects the respiratory health of cancer survivors can identify a potentially modifiable risk factor contributing to respiratory problems in this population. The identification of the potential for environmental factors to exacerbate cancer late effects may increase in importance as this population grows in number and age [48].

The role of air pollution on the health of cancer survivors has been largely understudied. We found that PM_2.5_ was associated with a two-fold increase in the odds for hospitalizations caused by respiratory infection. Chemotherapy-treated survivors had a significantly higher risk for respiratory hospitalizations attributed to PM_2.5_ than a cancer-free population sample. Certain chemotherapies are linked to short- and long-term immunosuppression [49]. Although most patients recover immune function within six months of treatment cessation, some patients do not recover full immune function [18,50]. These survivors report persistently low T-cell counts, reducing their response to infection [49]. Long after therapy ceases, cancer survivors display an increased risk for pneumonia and other respiratory infections when compared to siblings [18]. The immunosuppressive effects of PM_2.5_ are well documented and PM_2.5_ is a primary risk factor for respiratory infections in children through immune suppression [51]. Our findings support the hypothesis that immunosuppression from chemotherapy may be further exacerbated by PM_2.5_ to result in respiratory infection.

The odds of PM_2.5_-related respiratory ED visits and respiratory infections were highest among survivors diagnosed at age ≤3 years compared to survivors of older age groups. Of survivors diagnosed at age ≤3 years, 27% of our sample were diagnosed with leukemia. Leukemia is the most commonly diagnosed childhood cancer and requires chemotherapy treatment ranging from two to three years [52]. As such, survivors are at risk for long-term health effects that may increase their risk for PM_2.5_-related respiratory illness. Exposure to certain chemotherapies at a young age is linked to long-term pulmonary dysfunction and diffusion abnormalities in up to 65% of childhood cancer survivors [15,53]. Pulmonary dysfunction and immunosuppression related to chemotherapy [49] may explain the increase in risk for respiratory infection seen among survivors diagnosed at age ≤3 years in this study. Further follow-up is needed to confirm the association between leukemia and post-treatment PM_2.5_-related respiratory problems.

A previous study of this cohort reports a higher risk for mortality due to infection among Hispanic cancer survivors [54]. In the current report, we found a significant association between PM_2.5_ and respiratory infection events from a combined group of Hispanic/Other ethnicity survivors, the majority of whom were Hispanic. Further investigation of the influence of air pollution on differences in infection-related outcomes between Hispanic and Non-Hispanic White survivors is needed. As racial and ethnic minorities are more likely to live in neighborhoods with higher levels of air pollution [55,56], the impact of air pollution on cancer survivors who are racial and ethnic minorities may be of interest for investigation in larger minority populations.

We found different results in the PM_2.5_-associated odds between the hospitalizations and the ED visits. An earlier study of this cohort found that cancer survivors had a significantly increased rate of ED visits compared to the general population, with a high rate of use for respiratory infection [57]. Nationwide, infection is a leading cause of ED visits among childhood cancer survivors [58]. We did not find significant associations between PM_2.5_ and ED visits for respiratory infections, but we did find a significant association between PM_2.5_ and hospitalizations due to respiratory infection. This could be due to the different methods by which ED visits and hospitalizations are accessed by the public and cancer survivors. Emergency departments are available to the public and as a consequence, ED admission does not always equate to urgent needs or severe disease [59,60]. In contrast, hospitalizations require a clinician or ED referral and may indicate more severe disease or conditions [32,60].

In our analyses, the majority of our hospitalizations and ED visits occurred when the 24-h PM_2.5_ levels were below the 24-h standard (35.4 µg/m^3^) considered acceptable by both national and statewide agencies [45,61]. In Utah, winter inversions raise the PM_2.5_ above the 35.4 µg/m^3^ standard. Although we did not identify the p-value for the comparisons by NAAQS standard, we found that the odds of an ED visit when the PM_2.5_ was ≥35.4 µg/m^3^ and >25.0 µg/m^3^ were slightly lower than the odds at <35.4 µg/m^3^ and <25.0 µg/m^3^. Odds ratios for hospitalization were positive and significant at PM_2.5_ <35.4 µg/m^3^ and <25.0 µg/m^3^. Although needing further verification, our data suggest that the inversions with PM_2.5_ levels ≥35.4 µg/m^3^ are not the only driver behind these hospitalizations and that lower levels may be more relevant to this population. The general public in Utah is given alerts via the mainstream media on days with poor air quality defined by PM_2.5_ >35.4 µg/m^3^. These media alerts occur most often in the winter when inversions often raise the PM_2.5_ higher than 35.4 µg/m^3^ and the PM_2.5_ pollution can be seen by the naked eye. Together with the cold weather, these factors likely persuade some of the public to limit their PM_2.5_ exposure by avoiding outdoor activity. Our results suggest that cancer survivors may need to be specifically alerted if the PM_2.5_ are lower than the current 24-h standard of 35.4 µg/m^3^. The identification of vulnerable populations is key to updating and improving air quality policies. Due to the severe pulmonary damage from chemotherapy, childhood cancer survivors may be one such population that can help inform future air quality policies. 

Our findings have particular importance for cancer survivor follow-up care and public health guidelines. The Children’s Oncology Group (COG) provides risk-based guidelines for the lifelong care of childhood cancer survivors [62]. Current recommendations for respiratory health include the avoidance of tobacco products and secondhand tobacco smoke, another environmental contaminant [62]. Air pollution is not considered by COG, medical providers, or other follow-up care guidelines as an environmental contaminant for cancer survivors to avoid. Moreover, cancer survivors are not included in national public health guidelines that address air pollution. The Centers for Disease Control and Prevention specifically recommends that children with asthma avoid outdoor activities during elevated pollution days, but does not include guidance for cancer survivors and other groups with treatment-related morbidities [63]. Our results suggest that survivorship and public health guidelines may need to be revised to consider cancer survivors and persons with treatment-related respiratory complications as potential vulnerable populations to air pollution, although more follow-up and a larger sample size are needed to confirm results.

### Limitations

While our sample size is limited, the confidence intervals we report are relatively narrow and provide support for the stability of our findings. We did not have information about specific chemotherapy type and radiation field. We did not have data on smoking or secondhand smoke, which could act as an effect modifier. As this is a case-crossover study, smoking should not confound our estimates. We limited our analyses to events occurring before the age of 40 to ensure that the events in the survivor and cancer-free sample group occurred during the same time frame and at similar ages. This restriction could have induced bias but in sensitivity analyses we found no significant differences between the estimates in the cancer survivor events when all ages were included as 94% of events in the cancer survivor sample and 90% of events in the cancer-free sample occurred at age <40 years. Including daily PM_2.5_ estimated from county-level PM_2.5_ and from regression models using PM_10_ may introduce exposure misclassification biasing our estimates towards the null, but our sensitivity analysis did not detect major differences with or without the estimated PM_2.5_ values. We did see improvements in power thus the estimated PM_2.5_ days remained in the analysis.

As the subjects who contributed events to the cancer-free sample in this study were pre-screened for absence of any cancer diagnosis, our cancer-free sample may be healthier than the general population. Another reason for the difference is that the number of events among the cancer-free sample may not have provided enough power to approximate the odds in the general population. Increasing the number of events included from the cancer-free sample, similar to increasing the number of controls in a case-control study, may ensure approximation of the general population odds ratio [64].

In addition, the odds reported for the cancer-free sample in this study are lower than the odds reported in the general pediatric Utah population [20]. Our odds ratios for the cancer-free sample are 0.84 for hospitalizations and 0.82 for all respiratory infections. An investigation by Horne et al in the general Utah pediatric, adolescent, and young adult populations reported significant odds ratios of 1.02 to 1.22 per 10-unit increases in PM_2.5_ and increased risk for acute lower respiratory infections [20], depending on the age group studied and the exposure window. Although not directly comparable with our study, the odds ratios reported for our cancer survivors are higher than the odds ratios reported for the general population by Horne et al, especially for hospitalizations. Of particular interest were the odds ratios we report of 2.09 for the PM_2.5_-associated odds of hospitalization due to respiratory infection among all survivors, and 2.03 for respiratory hospitalization among chemotherapy-treated survivors. Continued follow up is needed to confirm our results as well as future studies that enlarge the number of events available for analysis.

## 5. Conclusions

Healthy survivorship is a priority for the growing number of survivors of childhood cancers in the United States [65]. Although medical interventions are critical to childhood cancers survivors’ long-term care [66], this study provides evidence that the respiratory morbidity of cancer survivors may be impacted by the environment in which they reside [48]. Since childhood cancer survivors often complete therapy before adolescence, most have decades of life remaining after treatment ceases. To prevent future respiratory morbidity, childhood cancer survivors should take action to avoid air pollution exposure, and survivorship guidelines and health agencies should consider revising current policies and guidelines to include cancer survivors as a vulnerable population. As respiratory morbidity is a leading cause of non-cancer death in this population [13,14,17], providing adequate guidelines for preserving cancer survivors’ health and identifying post-treatment factors that affect survivors’ health may be key to preventing future morbidity.

## Figures and Tables

**Table 1:** Event and control days, number of hospital and emergency department (ED) event days, and mean PM_2.5_ and temperature included in the case-crossover study.

Causes of Ddmission	Event Days	Control Days	Total Days
	*n*	*n*	*n*
**Total number of days**	335	1156	1491
Total hospitalizations	68	233	301
Total ED visits	267	923	1190
**Respiratory infections**			
Hospitalizations	41	141	182
ED visits	175	606	781
**Respiratory disease**			
Hospitalizations	25	85	110
ED visits	64	222	286
**Asthma**			
Hospitalizations	^	7	9
ED visits	28	95	123
	Mean	Range	
Average 3-day PM_2.5_ (µg/m^3^)	10.0	1.15–68.7	
Average 3-day temperature (°C)	8.4	−22.0 to 36.1	

^ Less than 5 events.

**Table 2:** Counts of survivors and event days by demographic and clinical factors.

Sample Characteristics	Survivors		All Event Days		Hospitalizations		ED Visits	
	*n*	%	*n*	%	*n*	%	*n*	%
Total	185		335		68		267	
Sex								
Female	75	40.5	136	40.6	27	39.7	109	40.8
Male	110	59.5	199	59.4	41	60.3	158	59.2
Race/ethnicity ^1^								
Hispanic/other	30	16.2	59	17.6	12	17.7	47	17.6
White, Non-Hispanic	154	83.2	275	82.1	56	82.4	219	82.0
Cancer diagnosis								
Leukemia	48	26.0	88	26.3	12	17.7	76	28.5
Lymphomas	43	23.2	91	27.2	19	27.9	72	27.0
CNS neoplasms	38	20.5	76	22.7	16	23.5	60	22.5
Sarcomas/bone tumors	42	22.7	61	18.2	15	22.1	46	17.2
Other solid tumors	14	7.6	19	5.7	6	8.8	13	4.9
Age at diagnosis (years)								
0 to 3	52	28.1	97	29.0	18	26.5	79	29.6
4 to 10	42	22.7	75	22.4	15	22.1	60	22.5
11 to 18	47	25.4	75	22.4	11	16.2	64	24.0
19 to 25	44	23.8	88	26.3	24	35.3	64	24.0
Years since diagnosis								
5 to 9	115	62.2	188	56.1	44	64.7	144	53.9
10 to 29	70	37.8	147	43.9	24	35.3	123	46.1
Chemotherapy								
No	65	35.1	135	40.3	23	33.8	112	42.0
Yes	120	64.9	200	59.7	45	66.2	155	58.1

^1^ Missing event with race/ethnicity = 1.

**Table 3:** Odds ratios and 95% confidence intervals (CI) for the main and stratified effects of a 10 µg/m^3^ increase in PM_2.5_ with respiratory hospitalizations among survivors of childhood cancers.

Main and Stratified Hospitalization Models	Odds Ratio	95% CI
Main effect	1.84 *	1.13–3.00
Stratified Models		
Cause of admission		
Respiratory infection	2.09 *	1.06–4.14
Respiratory disease	1.68	0.81–3.40
Race/ethnicity		
Hispanic	2.22	0.93–5.27
White, Non-Hispanic	1.64	0.88–3.05
Previous chemotherapy		
No	1.35	0.50–3.66
Yes	2.03 *	1.14–3.61
Age at diagnosis (years)		
0 to 3	2.24	0.94–5.36
4 to 10	2.08	0.83–5.21
11 to 18	1.09	0.25–4.71
19 to 26	1.75	0.56–5.43
Years since diagnosis		
5 to 9	1.76 *	1.03–3.01
10 to 29	2.29	0.70–7.54
NAAQS standard^1^		
<35.4 µg/m^3^	1.79	0.99–3.26
Below NAAQS standard ^1^		
<25.0 µg/m^3^	2.48 *	1.16–5.05

Models controlled for temperature; * Significant 95% CI; ^1^ OR and 95% CI for ≥35.4 and ≥25.0 unstable due to small numbers.

**Table 4:** Odds ratios and 95% confidence intervals (CI) for the main and stratified effects of a 10 µg/m^3^ increase in PM_2.5_ with respiratory emergency department (ED) visits among survivors of childhood cancers.

Main and Stratified ED Visit Models	Odds Ratio	95% CI
Main effect	1.04	0.86–1.26
Stratified Models		
Cause of admission		
Respiratory infection	1.02	0.80–1.29
Respiratory disease	1.01	0.69–1.49
Asthma	1.17	0.74–1.84
Race/ethnicity		
Hispanic/Other	1.28	0.86–1.89
White, Non-Hispanic	0.98	0.79–1.22
Previous chemotherapy		
No	0.86	0.62–1.20
Yes	1.16	0.92–1.45
Age at diagnosis (years)		
0 to 3	1.58 *	1.09–2.30
4 to 10	1.04	0.72–1.50
11 to 18	0.71	0.44–1.14
19 to 26	0.86	0.55–1.34
Years since diagnosis		
5 to 9	0.97	0.74–1.29
10 to 29	1.11	0.86–1.44
NAAQS standard		
≥35.4 µg/m^3^	1.11	0.34–3.62
<35.4 ug/m^3^	1.15	0.88–1.51
Below NAAQS standard		
≥25.0 µg/m^3^	1.07	0.52–2.18
<25.0 µg/m^3^	1.21	0.84–1.73

Models controlled for temperature; * Significant 95% CI.

**Table 5:** Odds ratios and 95% confidence intervals (CI) for the main and stratified effects of a 10 µg/m^3^ increase in PM_2.5_ with respiratory infections among survivors of childhood cancers.

Main and Stratified Respiratory Infection Models	Odds Ratio	95% CI
Main effect	1.08	0.86–1.36
Stratified models		
Race/ethnicity		
Hispanic/Other	1.61 *	1.04–2.49
White, Non-Hispanic	0.93	0.71–1.23
Previous chemotherapy		
No	0.89	0.62–1.29
Yes	1.24	0.92–1.67
Age at diagnosis (years)		
0 to 3	1.63 *	1.03–2.58
4 to 10	1.08	0.66–1.77
11 to 18	0.79	0.47–1.33
19 to 26	0.96	0.60–1.56

Models controlled for temperature; * Significant 95% CI.

## Appendix A

**Table A1:** Adjusted odds ratios and 95% confidence intervals (CI) for the association of a 10-unit increase in PM_2.5_ with any respiratory healthcare encounter among survivors of childhood cancers.

Main and Stratified Models	OR	95% CI
All healthcare encounters	1.10	0.92–1.31
Stratified Models		
Cause of admission		
Respiratory infection	1.08	0.86–1.36
Respiratory disease	1.08	0.76–1.54
Asthma	1.19	0.75–1.87
Race/ethnicity		
Hispanic/Other	1.43	0.99–2.05
White, Non-Hispanic	1.01	0.83–1.24
Cancer diagnosis		
Leukemia	1.38	0.98–1.96
Lymphomas	0.84	0.58–1.21
CNS neoplasms	0.83	0.54–1.30
Sarcoma/bone tumors	1.29	0.89–1.88
Other solid tumors	2.23	0.80–6.21
Previous chemotherapy		
No	0.87	0.63–1.20
Yes	1.23	0.99–1.53
Age at diagnosis (years)		
0 to 3	1.65 *	1.17–2.34
4 to 10	1.13	0.80–1.59
11 to 18	0.73	0.47–1.14
19 to 26	0.89	0.59–1.34
Years since diagnosis		
5 to 9	1.09	0.85–1.39
≥10	1.12	0.87–1.44

Models controlled for temperature; * Significant 95% CI.
